# The effect of albumin and hemoglobin levels on the prognosis of early-stage cervical cancer: a prospective, single-center–based cohort study

**DOI:** 10.1186/s12905-023-02713-5

**Published:** 2023-10-24

**Authors:** Xinmei Wang, Juan Xu, Hongyuan Zhang, Pengpeng Qu

**Affiliations:** Department of Gynecological Oncology, Tianjin Central Hospital of Gynecology and Obstetrics, Tianjin Central Hospital of Gynecology and Obstetrics, No 156, Nankaisan Road, Tianjin, 300100 China

**Keywords:** Albumin, Anemia, Cervical cancer, Hemoglobin, Hypoalbuminemia, Malnutrition, Prognosis

## Abstract

**Background:**

Serum albumin (ALB) and hemoglobin (HGB) are important serum biochemical indices of the nutritional status of patients and are associated with cancer development. We investigated the relationship between ALB and HGB levels and clinicopathologic characteristics of early-stage cervical cancer to determine the influence of ALB and HGB on the prognosis of early-stage cervical cancer.

**Methods:**

The clinical data of 560 patients with International Federation of Gynaecology and Obstetrics (FIGO, 2009) stage IA1-IIA2 cervical cancer from January 2005 to December 2010 were retrospectively analyzed. The relationship between serum ALB and HGB levels and clinicopathological characteristics of patients were analyzed. The patients were followed-up for 12–138 months. The effects of ALB and HGB levels on the prognosis were analyzed by Cox regression, log-rank test, and the Kaplan–Meier method.

**Results:**

The rate of patients with pelvic lymph node metastasis (PLNM), tumor diameter ≥ 4 cm, lymphovascular space invasion (LVSI), and deep stromal invasion was significantly higher in the anemia and hypoalbuminemia group than in the normal group (*P* < 0.05). The progression-free survival (PFS) and overall survival (OS) of patients in the hypoalbuminemia group and anemia group were significantly lower than that of the normal group (*P* < 0.05). FIGO stage, tumor diameter, PLNM, depth of stromal invasion, LVSI, the levels of ALB and HGB were risk factors for the prognosis of cervical cancer patients (*P* < 0.05).

**Conclusion:**

Patients with hypoproteinemia and anemia in early-stage cervical cancer are more likely to have higher tumor stage, larger tumor size, PLNM, LVSI, and deep stromal invasion. In addition, patients with hypoproteinemia and anemia have a poorer prognosis than those without the condition. Therefore, it is of great significance to detect the ALB and HGB levels of patients and improve the nutritional status of patients in a timely manner for better prognosis of cervical cancer.

## Introduction

Although cervical cancer is largely prevented through routine screening for HPV and vaccination, the latest cancer statistics show that it remains one of the major threats to women's health worldwide [1]. Clinical treatment of cervical cancer usually includes a variety of treatment methods, and surgical resection is still the main treatment for patients with FIGO stage IA1-IIA2 [2]. At present, clinicopathological factors such as tumor size, PLNM, LVSI, histological type and tumor grade have been confirmed to be the main risk factors affecting the prognosis of cervical cancer [3]. However, detailed information about these factors often needs to be obtained after surgical treatment, which limits their clinical application to some extent. Therefore, it is urgent to find effective preoperative prognostic indexes for prognosis assessment and individualized treatment of patients with cervical cancer.

The incidence of malnutrition in patients with malignant tumors is 40–80% [4]. Malnutrition can directly affect the therapeutic outcomes of cancer treatment, reduce the patient’s quality of life, cause organ dysfunction, and increase complications [5]. Serum albumin (ALB) and hemoglobin (HGB) are important serum biochemical indices of the nutritional status of patients and are associated with cancer development [6]. Nutritional status is closely related to tumor inflammatory response [7]. Cancer-associated inflammatory mediators promote tumor growth, invasion, metastasis, and angiogenesis [8]. Anemia and hypoalbuminemia affect the immune tolerance environment of tumor tissues and can activate proinflammatory-related pathways, thereby affecting the occurrence and development of tumors and patients’ prognosis [9].

Thus far, there are few studies on the relationship between ALB and HGB level and prognosis of early cervical cancer. Therefore, in this study, we retrospectively analyzed the clinical data of 560 early-stage cervical cancer patients admitted to our hospital between January 2005 and December 2010. The serum ALB and HGB levels of patients were noted, and the relationship of ALB and HGB levels with the clinicopathological characteristics of patients and their impact on prognosis was assessed.

## Methods

### Patients

The clinical data of 560 patients with International Federation of Gynaecology and Obstetrics (FIGO, 2009) stage IA1-IIA2 cervical cancer admitted to Tianjin Central Hospital of Gynecology and Obstetrics between January 2005 and December 2010 were retrospectively analyzed using the hospital medical records.

The inclusion criteria were: 1) patients with squamous, adenocarcinoma, or adenosquamous carcinoma of the cervix, 2) those with FIGO (2009) stage IA1-IIA2 cervical cancer; and 3) those who received primary radical surgical treatment comprising transabdominal radical hysterectomy and bilateral pelvic lymphadenectomy. The exclusion criteria were as follows: 1) patients with small-cell neuroendocrine carcinoma, cervical sarcoma, cervical lymphoma, cervical melanoma, and other cervical nonepithelial tumors, 2) preoperative metastatic cervical cancer, 3) patients who received radiotherapy or neoadjuvant chemotherapy before surgery, and 4) cases complicated by malignant tumors in other organ systems. 5) Patients with dysfunction of the liver, kidney, and other important organs, 6) Patients with other diseases affecting HGB and ALB levels.

### Diagnostic criteria for hypoproteinemia and anemia

Peripheral venous blood was collected from all women. The blood was collected before the operation, and the serum levels of ALB and total protein were detected by colorimetry using an automatic biochemical analyzer (Beckmann, USA). In addition, the HGB level was detected by colorimetry using an XN-9000 automatic hematology analyzer (Nisimori Mica, Japan). HGB < 110 g/L was diagnosed as anemia, and ALB < 35 g/L was diagnosed as hypoalbuminemia.

### Pathological diagnosis

Two gynecologic pathologists reviewed the pathological slides of each patient to confirm tumor diameter, pelvic lymph node metastasis (LNM), lymphovascular space invasion (LVSI), depth of cervical stromal invasion, parametrial invasion, and pathological type. The largest dimension was recorded as tumor diameter. Deep stromal invasion was defined as the depth of cervical stromal invasion ≥ 1/2.

### Postoperative adjuvant therapy

Patients with any of the “High risk factors” were recommended for further imaging to understand metastases at other sites, followed by external pelvic irradiation plus platinum-containing concurrent chemotherapy and/or brachytherapy. High risk factors include positive lymph nodes, positive incisal margins and parametrial invasion. Patients with Medium risk factors were supplemented with external pelvic irradiation and/or platinum-containing concurrent chemotherapy according to Sedlis criteria. Medium risk factors include tumor size, interstitial infiltration, and positive lymphatic vascular space, However, the medium risk factors are not limited to Sedlis criteria, but also include adenocarcinoma and tumor proximity to the incisal margin.

### Follow-up

All patients were followed-up immediately after surgery. The follow-up deadline was April 1, 2021. The follow-up period was 12–138 months (median: 117.83 months) and the follow-up loss rate was 1.43%. All patients were followed-up in the outpatient clinic or over the telephone, once every 3 months within 2 years after the end of treatment, once every 6 months thereafter, and once per year after 5 years. The follow-up included reexamination of routine blood parameters, blood biochemistry, tumor markers, chest CT, abdominal enhanced CT, and abdominal color ultrasound.

Progression-free survival (PFS) refers to the time from the initiation of follow-up to the appearance of objective tumor progression or death. Overall survival (OS) refers to the time from the start of the follow-up to the time of patient’s death from any cause. Disease progression was defined as an increase of at least ≥ 20% in the sum of maximum diameters of the target lesion or the appearance of a new lesion.

Patient anonymity was preserved as the data were collected from the hospital’s electronic medical records. In addition, the research ethics committee of Tianjin Central Hospital of Gynecology and Obstetrics waived the requirement for ethics approval and informed consent, because the study used previously stored data.

### Statistical analysis

SPSS version 28.0 (IBM Corporation, Armonk, NY, USA) was used for all statistical analyses. All tests were two-sided, with the significance threshold at *P* < 0.05. The chi-square test analyzed the relationship between ALB and HGB levels with clinicopathological characteristics. The log-rank test was used for single-factor analysis of the influence of preoperative ALB and HGB levels on the long-term postoperative survival of patients. Finally, the Kaplan–Meier method was used to plot survival curves. The effects of albumin and hemoglobin levels on the prognosis were analyzed by Cox regression.

## Results

### Association between ALB level and clinicopathological characteristics of cervical cancer patients

The patients were divided into two groups according to the level of ALB, namely the normal group (ALB ≥ 35 g/L, *n* = 344) and hypoalbuminemia group (ALB < 35 g/L, *n* = 216). Patients in both groups were further subdivided depending on age, FIGO stage, tumor diameter, PLNM, depth of stromal invasion, and LVSI. There were significant differences in FIGO stage, tumor diameter, incidence of PLNM, LVSI, and deep stromal invasion between the normal and hypoalbuminemia groups. The percentage of patients with FIGO stage II, tumor diameter ≥ 4 cm, PLNM, LVSI, and deep stromal invasion in the hypoalbuminemia group were significantly higher than in the normal group (*P* < 0.05). There was no significant difference in age between the two groups (*P* > 0. 05) (Table 1).

**Table 1:** Association between ALB level and clinicopathological characteristics

Characteristic	Patients (n)	Normal group (*n* = 344, 61.43%)	Hypoalbuminemia group (*n* = 216, 38.57%)	Chi-square	*P*-value
Age (years)					
< 50	312	216 (69.23)	96 (30.77)	2.263	0.133
≥ 50	248	128 (51.61)	120 (48.39)		
Tumor diameter (cm)					
< 4	408	304 (74.51)	104 (25.49)	24.646	< 0.001
≥ 4	152	40 (26.32)	112 (73.68)		
FIGO stage					
I	368	256 (69.57)	112 (30.43)	4.011	0.042
II	192	88 (45.83)	104 (54.17)		
PLNM					
Positive	216	72 (33.33)	144 (66.67)	17.85	< 0.001
Negative	344	272 (79.07)	72 (20.93)		
Deep stromal invasion					
No	296	216 (72.97)	80 (27.03)	4.417	0.035
Yes	264	128 (48.48)	136 (51.51)		
LVSI					
Negative	64	48 (75)	16 (25)	4.212	0.039
Positive	496	296 (59.68)	200 (40.32)		

*FIGO* International Federation of Gynaecology and Obstetrics, *LVSI* Lymphovascular space invasion, *PLNM* Pelvic lymph node metastasis

### Association between HGB level and clinicopathological characteristics of cervical cancer patients

The patients were divided into two groups according to the level of HGB, namely the normal group (HGB ≥ 110 g/L, *n* = 420) and anemia group (HGB < 110 g/L, *n* = 140). Patients in both groups were subdivided into two levels, depending on age, FIGO stage, PLNM, depth of stromal invasion, and LVSI. There were significant differences in FIGO stage, tumor diameter, incidence of PLNM, LVSI, and deep stromal invasion between the normal and anemia groups. The rate of patients with FIGO stage II, tumor diameter ≥ 4 cm, PLNM, LVSI, and deep stromal invasion in the anemia group were significantly higher than in the normal group (*P* < 0.05). There was no significant difference in age between the two groups (*P* > 0.05) (Table 2).

**Table 2:** Association between HGB level and clinicopathological characteristics

Characteristic	Patients (n)	Normal group (*n* = 420, 75%)	Anemia group (*n* = 140, 25%)	Chi-square	*P*-value
Age (years)					
< 50	397	304 (76.57)	93 (23.43)	0.336	0.562
≥ 50	163	116 (71.17)	47 (28.83)		
Tumor diameter (cm)					
< 4	171	108 (63.15)	63 (36.84)	5.916	0.021
≥ 4	389	312 (80.21)	77 (19.79)		
PLNM					
Positive	163	98 (60.12)	65 (39.88)	6.929	0.015
Negative	397	322 (81.11)	75 (18.89)		
FIGO stage					
I	402	332 (82.59)	70 (17.41)	9.238	0.002
II	158	88 (55.70)	70 (44.30)		
Deep stromal invasion					
No	362	306 (84.53)	56 (15.47)	4.52	0.031
Yes	198	114 (57.58)	84 (42.42)		
LVSI					
Negative	84	47 (55.95)	37 (44.05)	4.27	0.038
Positive	476	373 (78.36)	103 (21.64)		

*FIGO* International Federation of Gynaecology and Obstetrics, *LVSI* Lymphovascular space invasion, *PLNM* Pelvic lymph node metastasis

### Survival analysis

**Fig. 1:**
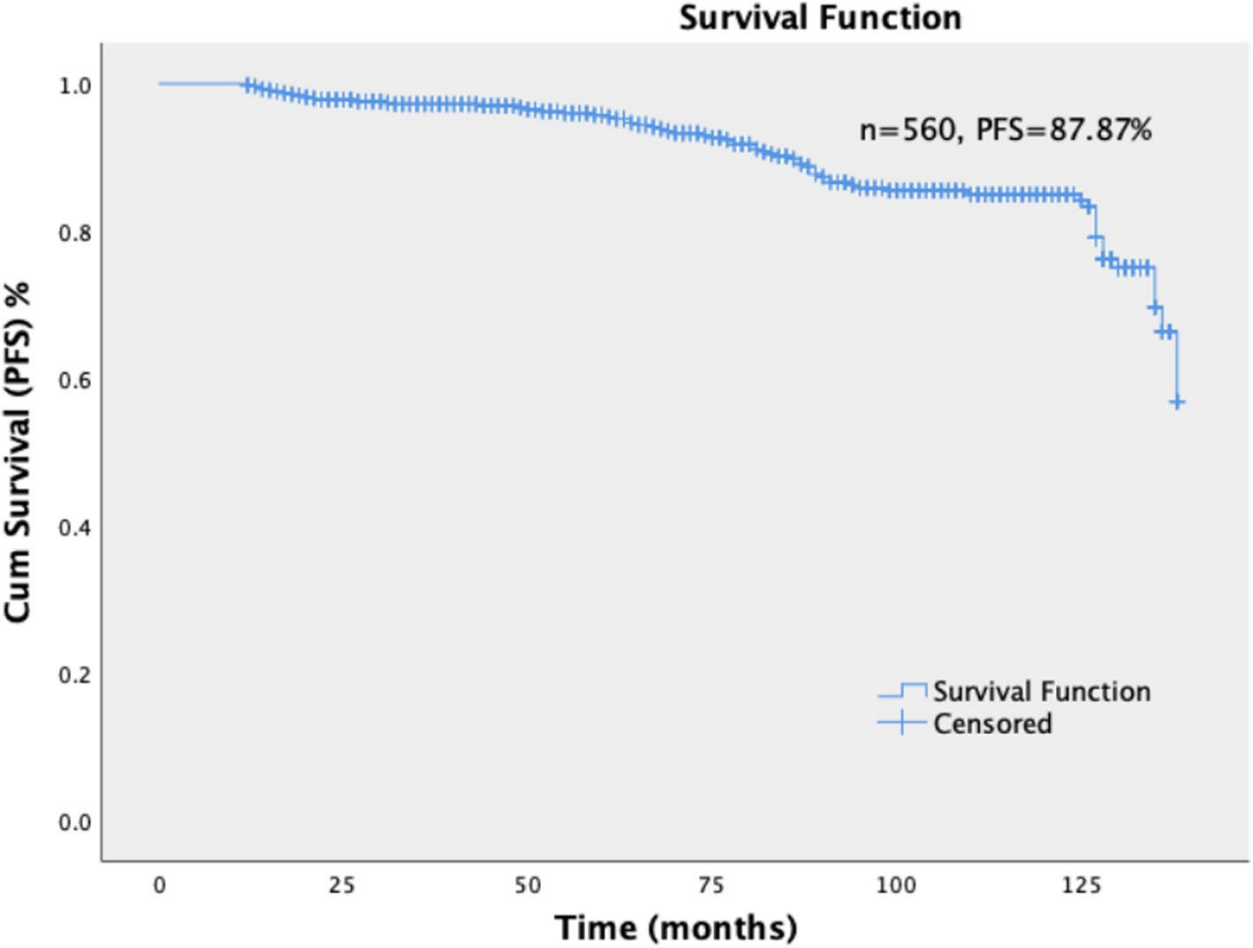
Kaplan–Meier curves for PFS of all patients. Five hundred sixty patients were followed up for 12–138 months; the median follow-up duration was 117.83 months. Kaplan–Meier curves for of all patients were included in the analysis; The PFS rate was 87.87%. PFS: Progression-free survival

**Fig. 2:**
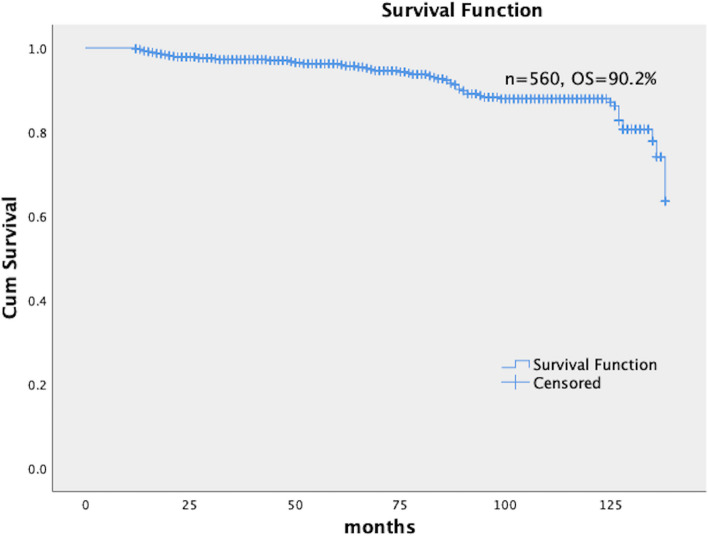
Kaplan–Meier curves for OS of all patients. Five hundred sixty patients were followed up for 12–138 months; the median follow-up duration was 117.83 months. Kaplan–Meier curves for of all patients were included in the analysis; The OS rate was 90.2%. OS: Overall survival

The patients were followed up for 12–138 months; the median follow-up duration was 117.83 months. Kaplan–Meier curves for PFS and OS of all patients were included in the analysis; The OS rate was 90.2%, and the PFS rate was 87.87% (Figs. 1 and 2).

### Relationship between ALB levels and prognosis of cervical cancer

**Fig. 3:**
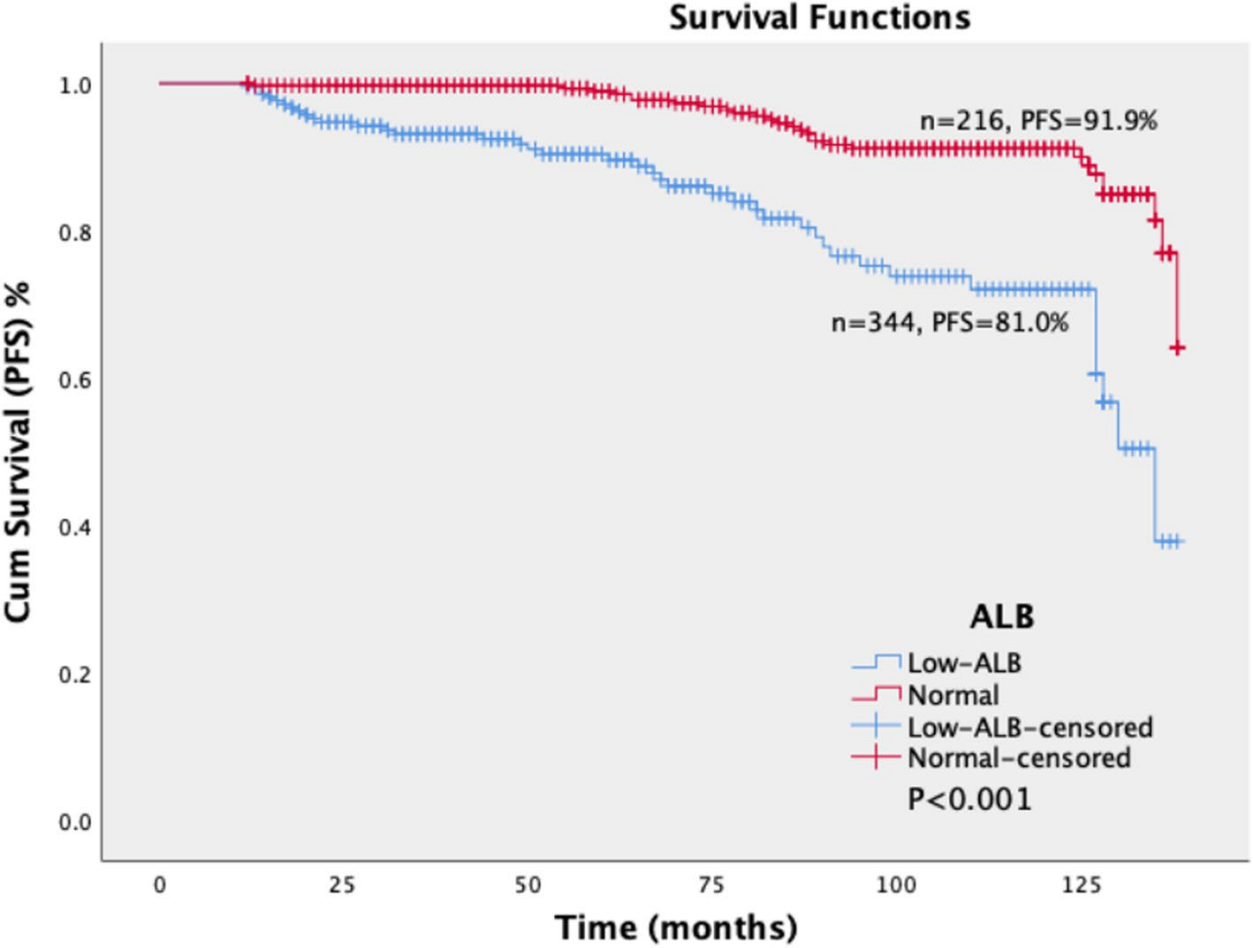
Survival curves of PFS in patients with different levels of ALB. The patients were divided into two groups according to the level of ALB, namely the normal group (ALB ≥ 35 g/L, *n* = 344) and hypoalbuminemia group (ALB < 35 g/L, *n* = 216). The log-rank test showed a significant difference in PFS the normal and hypoalbuminemia groups (*P* < 0.001). The PFS of the hypoalbuminemia group and normal group were 81.0% and 91.9%, respectively. The PFS of the hypoalbuminemia group were significantly lower than that of the normal group. ALB: Albumin; PFS: Progression-free survival

**Fig. 4:**
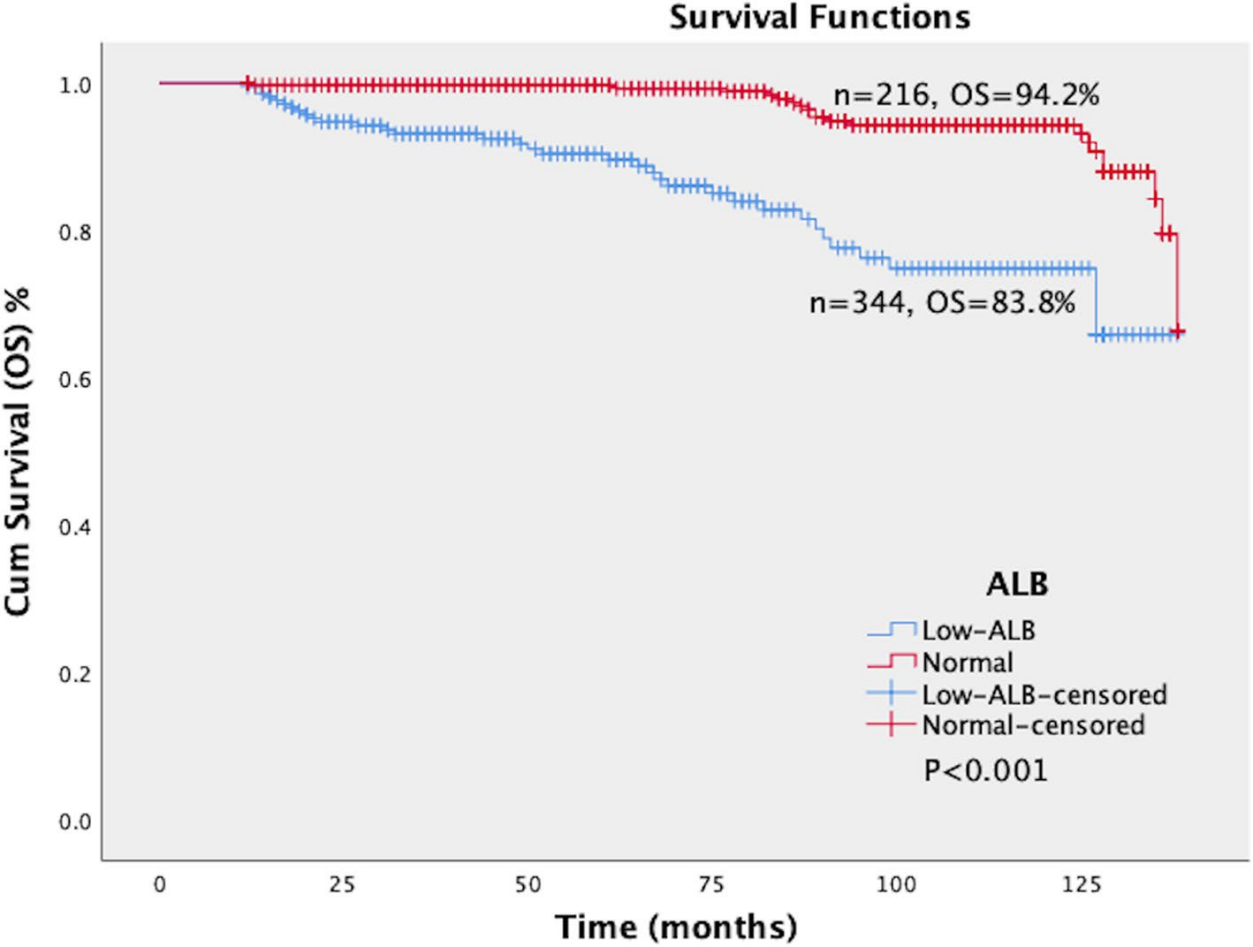
Survival curves of OS in patients with different levels of ALB. The patients were divided into two groups according to the level of ALB, namely the normal group (ALB ≥ 35 g/L, *n* = 344) and hypoalbuminemia group (ALB < 35 g/L, *n* = 216). The log-rank test showed a significant difference in OS between the normal and hypoalbuminemia groups (*P* < 0.001). The OS of the hypoalbuminemia group and the normal group were 83.8% and 94.2%, respectively. The OS of the hypoalbuminemia group were significantly lower than that of the normal group. ALB: Albumin; OS: Overall survival

The log-rank test showed a significant difference in PFS and OS between the normal and hypoalbuminemia groups (*P* < 0.001). The PFS of the hypoalbuminemia group and normal group were 81.0% and 91.9%, respectively. The OS of the hypoalbuminemia group and the normal group were 83.8% and 94.2%, respectively. The PFS and OS of the hypoalbuminemia group were significantly lower than that of the normal group (Figs. 3 and 4).

### Relationship between HGB levels and prognosis of cervical cancer

**Fig. 5:**
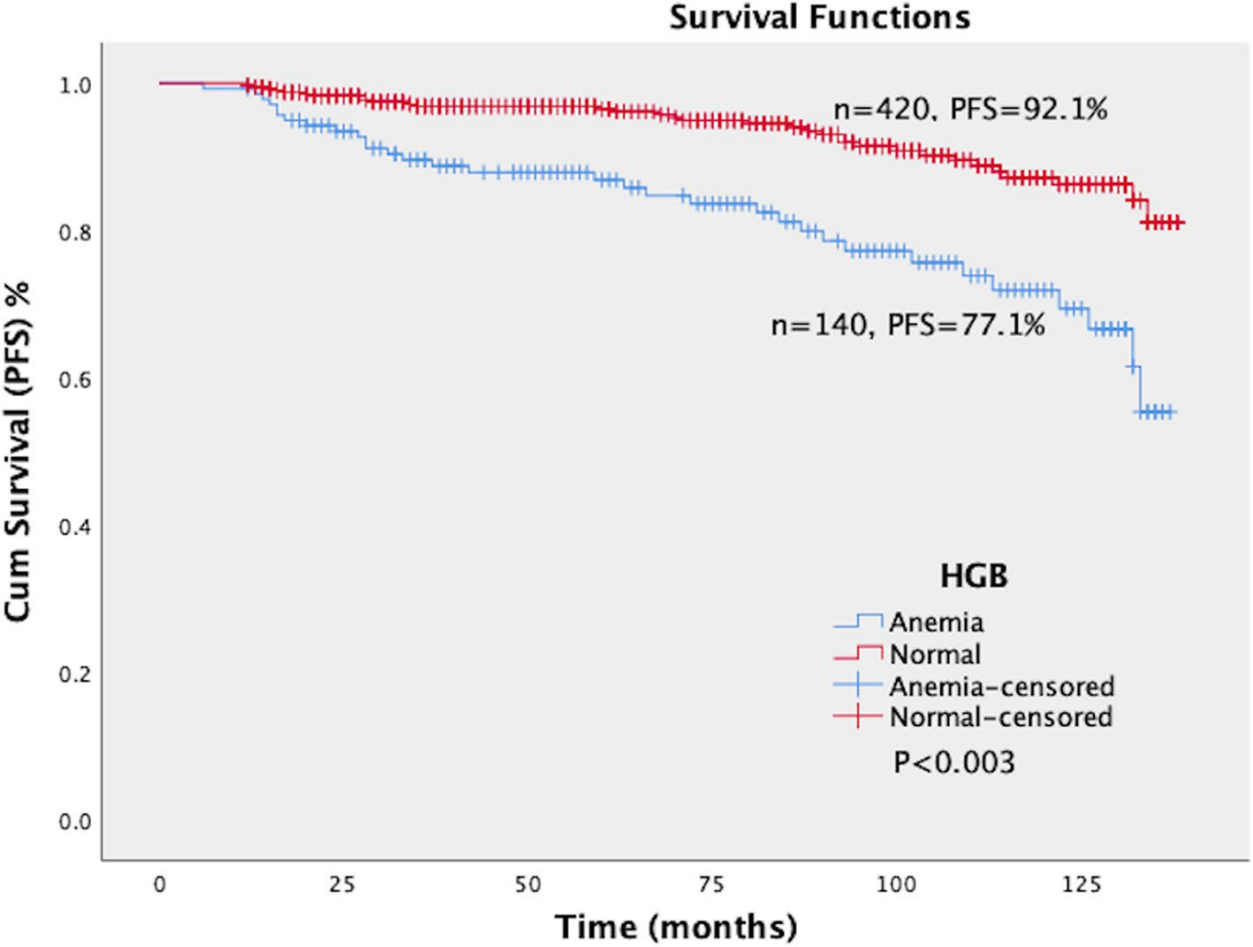
Survival curves of PFS in patients with different levels of HGB. The patients were divided into two groups according to the level of HGB, namely the normal group (HGB ≥ 110 g/L, *n* = 420) and anemia group (HGB < 110 g/L, *n* = 140). The log-rank test showed a significant difference in PFS between the normal and anemia groups (*P* < 0.003). The PFS of the anemia group and normal group were 77.1% and 92.1%, respectively. The PFS of the anemia group were significantly lower than that of the normal group. HGB: Hemoglobin; PFS: Progression-free survival

**Fig. 6:**
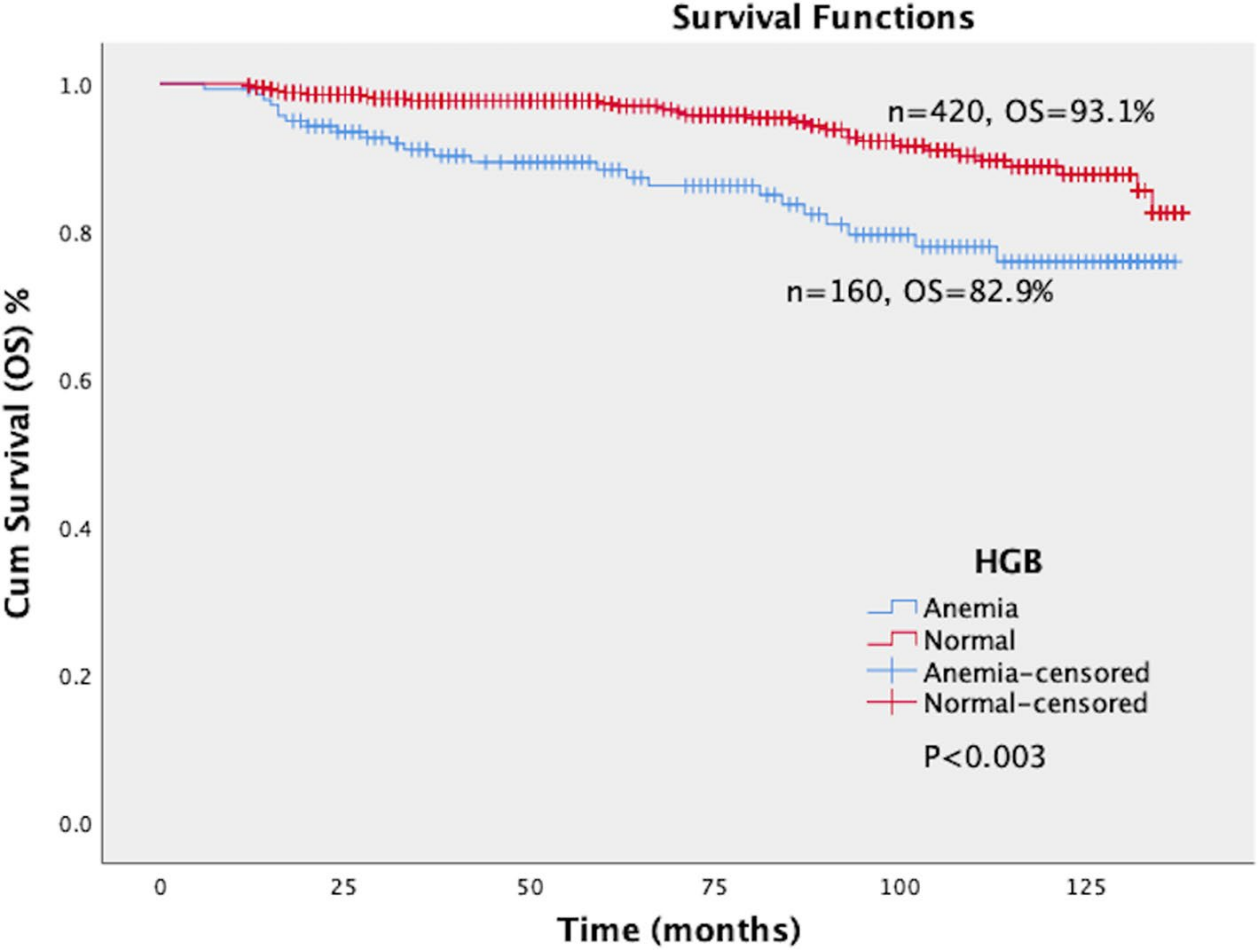
Survival curves of OS in patients with different levels of HGB. The patients were divided into two groups according to the level of HGB, namely the normal group (HGB ≥ 110 g/L, *n* = 420) and anemia group (HGB < 110 g/L, *n* = 140). The log-rank test showed a significant difference in OS between the normal and anemia groups (*P* < 0.003). The OS of the anemia group and normal group were 82.9% and 93.1%, respectively. The OS of the anemia group were significantly lower than that of the normal group. HGB: Hemoglobin; OS: Overall survival

The log-rank test showed a significant difference in PFS and OS between the normal and anemia groups (*P* < 0.003). The PFS of the anemia group and normal group were 77.1% and 92.1%, respectively. The OS of the anemia group and normal group were 82.9% and 93.1%, respectively. The PFS and OS of the anemia group were significantly lower than that of the normal group (Figs. 5 and 6).

### Cox regression analysis of prognostic factors of early-stage cervical cancer

Multivariate Cox regression analysis showed that FIGO stage, tumor diameter, PLNM, depth of stromal invasion, LVSI, the levels of ALB and HGB were risk factors for the prognosis of cervical cancer patients (*P* < 0.05) (Table 3).

**Table 3:** Cox regression analysis of prognostic factors of early-stage cervical cancer

Variable	*B*	SE	Wald	*P-*Value	OR(95% *CI)*
Age	-0.137	0.522	0.523	0.653	(0.316,1.127)
Tumor diameter	-0.831	0.271	5.871	0.019	(1.112,3.336)
PLNM	0.757	0.265	6.718	0.017	(2.132,3.586)
FIGO stage	0.684	0.311	9.038	0.002	(1.969,5.441)
Deep stromal invasion	1.411	0.306	4.661	0.035	(2.501,2.972)
LVSI	1.003	0.044	4.217	0.037	(1.114,3.346)
ALB	-0.831	0.271	5.871	0.019	(1.112,3.336)
HGB	0.757	0.265	6.718	0.017	(2.132,3.586)

*SE* Standard Error, *OR* Odds Ratio, *CI* Confidence Interval, *PLNM* pelvic lymph node metastasis, *FIGO* International Federation of Gynaecology and Obstetrics, *LVSI* Lymphovascular space invasion, *ALB* Albumin, *HGB* Hemoglobin

## Discussion

It is estimated that more than 300,000 of the 500,000 women diagnosed with cervical cancer worldwide die from the disease every year, among which tumor recurrence and metastasis are the main causes [10].

Recently, the use of blood routine, blood biochemical and other immunonutritional parameters to predict the recurrence and metastasis and long-term prognosis of patients with malignant tumors has attracted more and more attention.

Tumor recurrence, metastasis and poor prognosis depend not only on the biological characteristics of the tumor, but also on a variety of host-related factors. Recent studies have shown that the proliferation, invasion, metastasis and angiogenesis of tumor cells are closely related to the nutritional status of patients and the immune inflammatory response of the body [11, 12]. The nutritional and immune status of patients can be easily reflected by relevant indicators in the blood [13]. Serum ALB and HGB are common serum biochemical indicators, which can effectively reflect the nutritional status of patients with cervical cancer and are associated with the development of patients’ disease [6, 14].

Low ALB levels may indicate the existence of persistent systemic inflammation and immune system suppression, which are both important mechanisms underlying tumor occurrence and progression [15, 16]. Study [17] have shown that inflammation caused by HPV can lead to a decrease in the level of ALB, which was the result of systemic inflammatory response, and low albumin represents continuous systemic inflammatory response. It was also shown that albumin can inhibit systemic inflammation of tumor progression [18]. In this study, the results showed that the rate of patients with FIGO stage II, tumor diameter ≥ 4 cm, PLNM, LVSI, and deep stromal invasion in the hypoalbuminemia group were significantly higher than in the normal group (*P* < 0.05), which may be related to the reduced inflammatory inhibition ability of tumor.

Moreover, about 30% patients with cervical cancer were anemic before surgery, and severe anemia was a risk factor for mortality in such patients [19]. Preoperative transfusion and other strategies do not improve the prognosis of patients with cervical cancer, and patients with anemia have aggressive tumors, which further worsens the prognosis [20]. In this study, the rate of patients with FIGO stage II, tumor diameter ≥ 4 cm,P LNM, LVSI, and deep stromal invasion in the anemia group were significantly higher than in the normal group (*P* < 0.05). The results suggested that HGB levels may affect tumor progression, which was consistent with the results of previous studies. The reason may be that during the development of cervical cancer, protein catabolism increases and anabolism decreases, resulting in negative nitrogen balance and different degrees of HGB content reduction [21].

Studies have shown that ALB level, as an indicator of systemic inflammatory response, was helpful in judging the prognosis of solid tumors such as non-small cell lung cancer and hepatocellular carcinoma [15, 22]. Some studies have also shown that the C-reactive protein /ALB ratio was closely related to the prognosis of cervical cancer [16]. In addition to ALB, studies have also shown that HGB levels affect the prognosis of patients with cervical cancer [19, 23]. Many studies have shown that low preoperative HGB level was an independent risk factor for the prognosis of patients with cervical cancer [20, 24, 25]. In this study, the patients were followed-up for 12–138 months, and the PFS and OS of the hypoalbuminemia group and anemia group were both significantly lower than that of the normal group (*P* < 0.05), suggesting that the ALB and HGB level was closely related to the prognosis of cervical cancer patients, which was also consistent with previous studies. Multivariate Cox regression analysis showed that FIGO stage, tumor diameter, PLNM, depth of stromal invasion, LVSI, the levels of ALB and HGB were risk factors for the prognosis of cervical cancer patients(*P* < 0.05). The results further indicated that ALB and HGB levels were also important risk factors affecting the prognosis of patients with early cervical cancer in addition to traditional risk factors.

### Limitations

We were unable to evaluate all the variables potentially associated with the prognosis of early-Stage cervical cancer in a single study. Furthermore, as all included patients were from a single hospital, this might have reduced our results’ external validity. Hence, prospective studies with larger sample sizes and conducted across multi-centers are needed to validate our findings.

## Conclusions

Patients with hypoproteinemia and anemia in early-stage cervical cancer are more likely to have higher tumor stage; larger tumor size; and higher incidence of PLNM, LVSI, and deep stromal invasion. In addition, patients with hypoproteinemia and anemia in early-stage cervical cancer have a poorer prognosis than those without the condition. Therefore, it is vital to detect the ALB and HGB levels of patients and improve the nutritional status of patients in a timely manner to improve the prognosis of patients with cervical cancer.

## Data Availability

The datasets used and/or analysed during the current study available from the corresponding author on reasonable request.

## Abbreviations

ALB: Albumin
FIGO: International Federation of Gynaecology and Obstetrics
HGB: Hemoglobin
HPV: Human papillomavirus
LVSI: Lymphovascular space invasion
OS: Overall survival
PFS: Progression-free survival
PLNM: Pelvic lymph node metastasis

## References

[1] Sung H, Ferlay J, Siegel RL, Laversanne M, Soerjomataram I, Jemal A (2021). Global Cancer Statistics 2020: GLOBOCAN estimates of incidence and mortality worldwide for 36 cancers in 185 countries. CA Cancer J Clin.

[2] Bhatla N, Aoki D, Sharma DN, Sankaranarayanan R (2021). Cancer of the cervix uteri: 2021 update. Int J Gynaecol Obstet.

[3] Kong TW, Ryu HS, Kim SC, Enomoto T, Li J, Kim KH (2019). Asian Society of Gynecologic Oncology International Workshop 2018. J Gynecol Oncol.

[4] Torre LA, Bray F, Siegel RL, Ferlay J, Lortet-Tieulent J, Jemal A (2015). Global cancer statistics, 2012. CA Cancer J Clin.

[5] Donohoe CL, Ryan AM, Reynolds JV (2011). Cancer cachexia: mechanisms and clinical implications. Gastroenterol Res Pract.

[6] Sejima T, Iwamoto H, Masago T, Morizane S, Yao A, Isoyama T (2013). Low pre-operative levels of serum albumin predict lymph node metastases and ultimately correlate with a biochemical recurrence of prostate cancer in radical prostatectomy patients. Cent European J Urol.

[7] de Morais NG, da Costa TB, Pedrosa AL (2016). Effect of neonatal malnutrition on expression of nitric oxide synthase enzyme, production of free radicals and in vitro viability of alveolar macrophages infected with methicillin-sensitive and methicillin-resistant Staphylococcus aureus. Eur J Nutr.

[8] Ye J, Yin L, Xie P, Wu J, Huang J, Zhou G (2015). Antiproliferative effects and molecular mechanisms of troglitazone in human cervical cancer in vitro. Onco Targets Ther.

[9] Yoshino Y, Taguchi A, Shimizuguchi T (2019). A low albumin to globulin ratio with a high serum globulin level is a prognostic marker for poor survival in cervical cancer patients treated with radiation-based therapy. Int J Gynecol Cancer.

[10] Cohen PA, Jhingran A, Oaknin A, Denny L (2019). Cervical cancer. Lancet.

[11] Alwarawrah Y, Kiernan K, MacIver NJ (2018). Changes in nutritional status impact immune cell metabolism and function. Front Immunol.

[12] Zitvogel L, Pietrocola F, Kroemer G (2017). Nutrition, inflammation and cancer. Nat Immunol.

[13] Gangopadhyay A (2020). Prognostic nutritional index and clinical response in locally advanced cervical cancer. Nutr Cancer.

[14] Laky B, Janda M, Cleghorn G, Obermair A (2008). Comparison of different nutritional assessments and body-composition measurements in detecting malnutrition among gynecologic cancer patients. Am J Clin Nutr.

[15] Yang JR, Xu JY, Chen GC (2019). A prospective cohort study is that post-diagnostic C-reactive protein and ALB predict survival in Chinese patients with non-small cell lung cancer. Sci Rep.

[16] Zhang W, Liu K, Ye B, Liang W, Ren Y (2018). Pretreatment C-reactive protein/ALB ratio is associated with poor survival in stage IB-IIA cervical cancer patients. Cancer Med.

[17] He X, Li JP, Liu XH, Zhang JP, Zeng QY, Chen H (2018). Prognostic value of C-reactive protein/albumin ratio in predicting overall survival of Chinese cervical cancer patients overall survival: comparison among various inflammation based factors. J Cancer.

[18] Lv GY, An L, Sun XD, Hu YL, Sun DW (2018). Pretreatment albumin to globulin ratio can serve as a prognostic marker in human cancers: a meta-analysis. Clin Chim Acta.

[19] Lim S, Lee CM, Park JM, Jung SY, Lee KB (2014). An association between preoperative anemia and poor prognostic factors and decreased survival in early-stage cervical cancer patients. Obstet Gynecol Sci.

[20] Shin NR, Lee YY, Kim SH (2014). Prognostic value of pretreatment hemoglobin level in patients with early cervical cancer. Obstet Gynecol Sci.

[21] Uppal S, Al-Niaimi A, Rice LW, Rose SL, Kushner DM, Spencer RJ (2013). Preoperative hypoalbuminemia is an independent predictor of poor perioperative outcomes in women undergoing open surgery for gynecologic malignancies. Gynecol Oncol.

[22] Wen X, Yao M, Lu Y (2018). Integration of PreALB into Child-Pugh Classification Improves Prognosis Predicting Accuracy in HCC Patients Considering Curative Surgery. J Clin Transl Hepatol.

[23] Fuso L, Mazzola S, Marocco F (2005). Pretreatment serum hemoglobin level as a predictive factor of response to neoadjuvant chemotherapy in patients with locally advanced squamous cervical carcinoma: a preliminary report. Gynecol Oncol.

[24] Choi YS, Yi CM, Sin JI, Ye GW, Shin IH, Lee TS (2006). Impact of hemoglobin on survival of cervical carcinoma patients treated with concurrent chemoradiotherapy is dependent on lymph node metastasis findings by magnetic resonance imaging. Int J Gynecol Cancer.

[25] Li X, Tan C, Zhang W (2015). Correlation between platelet and hemoglobin levels and pathological characteristics and prognosis of early-stage squamous cervical carcinoma. Med Sci Monit.

